# Normative functional network localization of gray matter alterations in idiopathic generalized epilepsy

**DOI:** 10.3389/fneur.2026.1957963

**Published:** 2026-10-15

**Authors:** Xiang Wang, Zhi-Peng Zhang, Bo-Chao Zheng, Lu Liu, Gen-Di Wang, Hu-Cheng Yang, Liang-Liang Ma

**Affiliations:** 1 Department of Pediatrics, Binhai County People's Hospital, Yancheng, China; 2 Department of Pediatrics, Binhai Clinical College, Yangzhou University Medical College, Yancheng, China; 3 Department of CT, Hangjin Banner People's Hospital, Hangjin Banner, Inner Mongolia, China; 4 Department of Neurology, Affiliated Hospital 6 of Nantong University, Yancheng Third People’s Hospital, Yancheng, China; 5 Department of Radiology, Binhai Maternal and Child Health Hospital, Yancheng, China

**Keywords:** default mode network, functional connectivity network mapping, gray matter, idiopathic generalized epilepsy, subcortical network

## Abstract

**Objective:**

Idiopathic generalized epilepsy (IGE) has traditionally been considered a non-lesional epilepsy syndrome on conventional MRI, yet voxel-based morphometry (VBM) studies have reported subtle gray matter (GM) abnormalities across diverse brain regions. These findings remain spatially heterogeneous and difficult to reconcile. We aimed to characterize the normative functional connectivity patterns associated with reported GM alterations in IGE using functional connectivity network mapping (FCNM), and to explore whether these spatially heterogeneous findings show a reproducible pattern of observed network overlap.

**Methods:**

We systematically identified 18 whole-brain VBM studies of IGE, including 111 reported peak coordinates from 1,492 participants. Coordinates reflecting both increased and decreased GM were pooled in the primary analysis to characterize normative connectivity patterns associated with reported GM alterations irrespective of alteration direction. Using resting-state functional MRI (rs-fMRI) data from a normative discovery cohort (Human Connectome Project, *N* = 1,093), we mapped the functional connectivity of these GM coordinates to identify a convergent IGE-related network. The reproducibility of the observed spatial pattern was explored using an independent, cross-scanner validation cohort (Southwest University Adult Lifespan Dataset, *N* = 329) and two alternative seed sizes (1-mm and 7-mm radii).

**Results:**

Reported GM alterations in IGE were associated with an observed distributed normative functional connectivity pattern involving the bilateral middle temporal gyri, bilateral thalami, left superior frontal gyrus, and right putamen. In the HCP discovery analysis, this network showed the greatest observed overlap with the subcortical network (overlap proportion = 42.77%) and secondary correspondence with the default mode network (DMN; overlap proportion = 11.06%). Analysis using the independent SALD connectome showed a similar descriptive pattern, with the greatest observed overlap again involving the subcortical network (overlap proportion = 43.98%), whereas DMN correspondence was weaker.

**Conclusion:**

Reported GM alterations in IGE converge most robustly onto a subcortical normative functional network involving the thalamus and putamen, with additional DMN involvement observed primarily in the HCP discovery analysis. These findings provide preliminary, descriptive evidence that reported GM alterations in IGE are associated with a reproducible subcortical normative connectivity pattern across two connectome datasets, although the available analyses do not establish above-chance or disease-specific network convergence.

## Introduction

Idiopathic generalized epilepsy (IGE) refers to a group of common epilepsy syndromes that account for approximately 15–20% of all epilepsy cases (1). It encompasses several well-recognized syndromes, including childhood absence epilepsy (CAE), juvenile absence epilepsy (JAE), juvenile myoclonic epilepsy (JME), and epilepsy with generalized tonic–clonic seizures alone (GTCA) (2). Traditionally, IGE has been regarded as a “non-lesional” epilepsy syndrome, characterized by generalized spike–wave discharges in the absence of obvious structural abnormalities on routine MRI scans (2). However, the broader traditional perception of IGE as a benign disorder with limited long-term clinical impact has been challenged by accumulating evidence that individuals with IGE may experience persistent cognitive impairment (3), psychiatric comorbidities (4), and, in a substantial subset, inadequate seizure control or resistance to antiseizure medications (5). Collectively, these observations suggest that IGE should not be viewed solely as a benign disorder of transient generalized discharges, but rather as a condition with structural, cognitive, psychiatric, and network-level correlates.

Despite the absence of gross structural abnormalities on conventional MRI, patients with IGE exhibit gray matter (GM) alterations detectable with advanced neuroimaging techniques, notably voxel-based morphometry (VBM) (6). These abnormalities have been described across multiple cortical and subcortical regions, including the frontal, temporal, parietal, cingulate, insular, thalamic, basal ganglia, cerebellar, and limbic structures (7–18). However, the direction and location of these findings have varied substantially across studies, with some reporting GM increases, others reporting decreases (19–23), and several reporting no significant differences (24–28). This heterogeneity likely reflects small sample sizes, methodological variability in image acquisition and analysis, and clinical heterogeneity across cohorts. Such spatial inconsistency has hindered the identification of a consistent neuroanatomical signature for IGE, thereby obscuring our understanding of its fundamental pathophysiology.

Recently, neuroscience has increasingly shifted from a purely region-based framework toward a network-based understanding of brain disorders (29). Spatially heterogeneous abnormalities may be better reconciled by the observation that lesions or alterations at different anatomical locations can produce similar clinical manifestations when they are connected to a common functional network (30, 31). Motivated by this perspective, the localization of neuropsychiatric symptoms has transitioned from a predominantly region-based to a network-based paradigm. In this context, functional connectivity network mapping (FCNM) provides a framework for testing whether spatially heterogeneous brain abnormalities are connected to a common functional network. By combining reported abnormality coordinates with a large-scale normative connectome, FCNM estimates the normative functional networks connected to those locations, thereby testing whether spatially heterogeneous findings are embedded within a common network architecture (32). Importantly, this approach identifies normative connectivity profiles of reported abnormality sites rather than patient-specific functional connectivity abnormalities. FCNM has been applied successfully to map a variety of neurological and psychiatric symptoms onto common symptom-specific brain networks (33–41). However, few studies have applied FCNM to investigate whether reported GM alterations in IGE converge onto a common functional brain network.

Therefore, in this exploratory study, we applied FCNM to integrate findings from the existing VBM literature on IGE within a common functional-network framework. Specifically, we aimed to characterize the large-scale normative functional connectivity patterns showing the greatest observed spatial overlap with reported GM alterations in IGE. We hypothesized that reported GM alterations would show non-uniform and potentially reproducible observed overlap with a limited set of canonical networks. By doing so, we sought to reconcile previous inconsistencies in regional GM findings and to provide network-level insights that may inform future studies of network-based biomarkers and candidate therapeutic targets.

## Materials and methods

### Literature search and selection

Following the PRISMA guidelines, we conducted a comprehensive systematic search of the PubMed, Embase, and Web of Science databases to identify VBM studies on GM alterations in IGE, published up to 5 May 2026. The search included keywords such as (“gray matter”) AND (“epilepsy” OR “seizure” OR “JME” OR “juvenile myoclonic epilepsy” OR “GTCA OR “epilepsy with generalized tonic-clonic seizures alone” OR “AE” OR “CAE” OR “JAE” OR “childhood absence epilepsy” OR “juvenile absence epilepsy” OR “IGE” OR “idiopathic generalized epilepsy” OR “GGE” OR “Genetic Generalized Epilepsy”) AND (“voxel^*^” OR “VBM” OR “morphometry”). Additionally, we manually examined the bibliographies of relevant review articles and meta-analyses to identify any studies that might not have been included in the initial search strategy. A flow diagram of the study selection process is shown in Supplementary Figure S1.

Studies were included according to the following criteria: (a) published in an English-language peer-reviewed journal as an original article; (b) investigated VBM-based differences in GM density or volume between IGE patients and HCs; and (c) reported whole-brain VBM findings with three-dimensional peak coordinates in Talairach or Montreal Neurological Institute (MNI) space. For longitudinal studies, only baseline data were included. Exclusion criteria were as follows: (a) non-original studies (e.g., review, meta-analysis, meeting abstract); (b) no coordinate system reported; (c) only reported regions of interest (ROI) results; (d) all reported coordinates outside the GM mask; (e) studies reporting results based on small-volume correction; and (f) studies involving animal experiments. To avoid data duplication from overlapping patient samples across different publications, only the study reporting the largest sample size and most comprehensive information was retained for analysis. All coordinates from prior studies were converted to MNI space using established SDM-PSI 6.2.3 tools.^1^ The corresponding author of each included study was contacted via email when additional information was required. For studies reporting multiple independent IGE subgroups compared with independent or shared control groups, each patient-control comparison was treated as a separate contrast when non-overlapping patient samples were available. When multiple statistical thresholds or models were reported for the same cohort, the primary whole-brain corrected analysis was preferentially extracted. Two investigators (W. X and Z. Z. P) independently performed the literature search and selection and data extraction. Any discrepancies were resolved by discussion with another investigator (M. L. L) until consensus was reached.

For each included VBM study, we extracted the following information: first author and year, epilepsy subtype, number of subjects and number of female participants, mean age, disease duration, scanner field strength, statistical threshold, coordinate space, and number of reported foci. These variables were summarized descriptively to describe the clinical and methodological heterogeneity of the included studies. Because no standardized and validated risk-of-bias tool is currently available for coordinate-based VBM studies, we did not combine these items into an overall quality score.

### Discovery and validation datasets

Two independent cohorts were analyzed in this study: a discovery cohort from the Human Connectome Project (HCP) S1200 release, and an external validation cohort from the Southwest University Adult Lifespan Dataset (SALD) to evaluate cross-scanner generalizability. The discovery sample consisted of 1,093 healthy participants (594 females; mean age: 28.78 ± 3.69 years; range: 22–37 years), while the validation sample enrolled 329 healthy individuals (207 females; mean age: 37.81 ± 13.79 years; range: 18–60 years). Detailed demographic profiles for both cohorts are presented in Supplementary Table S1. High-resolution HCP neuroimaging data were acquired using a 3 T Siemens Connectome Skyra scanner, with comprehensive cohort characteristics documented in previous literature (42). The HCP study protocol received ethical approval from the Institutional Review Board of Washington University in St. Louis, and all participants provided written informed consent. For the SALD dataset, resting-state fMRI (rs-fMRI) scans were performed on a 3 T Siemens Trio system. Individuals were excluded based on the following criteria: MRI contraindications, active psychiatric or neurological disorders, use of psychotropic medication within the preceding 3 months, pregnancy, or a history of head trauma. Both datasets consisted of neurologically healthy participants and were used to estimate normative functional connectivity patterns, consistent with prior FCNM studies. We used adult normative connectomes because large-scale pediatric normative rs-fMRI datasets with comparable preprocessing and sample size were not available for the present analysis. The rs-fMRI acquisition parameters for both datasets are summarized in Supplementary Table S2.

### Rs-fMRI data acquisition and preprocessing

Resting-state fMRI (rs-fMRI) data preprocessing was performed using SPM12^2^ and the DPABI V8.1 toolbox.^3^ The SALD datasets are publicly accessible via their respective official repositories. To allow for magnetization equilibrium and participant adaptation, the first 10 volumes of each scan were discarded. The remaining volumes underwent slice-timing correction and head motion realignment. All participants met the motion threshold of maximum translation < 2.0 mm and rotation < 2.0°. To minimize non-neuronal noise, several nuisance covariates were regressed out, including linear drift, the Friston-24 motion parameters, white matter, cerebrospinal fluid, and global signals. Additionally, motion outliers (framewise displacement > 0.5 mm) were addressed by including them as spike regressors. Following nuisance regression, a band-pass filter (0.01–0.1 Hz) was applied. The functional images were then spatially normalized to the Montreal Neurological Institute (MNI) space using deformation fields derived from the segmentation of co-registered T1-weighted structural images. Finally, the normalized data were spatially smoothed using a 6-mm full-width at half-maximum (FWHM) isotropic Gaussian kernel.

The HCP rs-fMRI data were initially processed using the HCP minimal preprocessing pipeline, which encompasses EPI gradient distortion correction, motion correction, bias field correction, spatial normalization to MNI space, and ICA-FIX-based artifact removal (43, 44). Beyond these standard steps, further processing was performed using the DPABI toolbox (45). Specifically, we regressed out nuisance covariates, including the global, white matter, and cerebrospinal fluid signals. Subsequently, the data underwent temporal band-pass filtering (0.01–0.1 Hz) and were spatially smoothed using a 6-mm FWHM Gaussian kernel.

### FCNM analysis

The analysis concentrated on independent patient-control contrasts instead of individual coordinates or the complete literature-derived coordinate set. The GM alteration coordinates from each of the 18 contrasts were transformed into 4-mm-radius spherical regions and amalgamated into a unique seed mask for each contrast. Following the process, 18 contrast-specific seeds and their corresponding whole-brain functional connectivity maps were generated. Subsequently, the maps were binarized, and after voxel-wise FDR correction, overlaid across contrasts. The IGE-associated normative network was then defined as voxels demonstrating positive connectivity with at least 60% of the contrast-specific seeds (37, 39). Given the 18 contrast-level FCNM maps included in the analysis, the ≥60% criterion required overlap in at least 11 contrast-level maps for a voxel to be included in the final network. Using resting-state fMRI data from 1,093 healthy participants in the HCP cohort, whole-brain functional connectivity maps were calculated for each participant by correlating the mean time series of each contrast seed with the time series of every other brain voxel. Pearson correlation coefficients were subsequently transformed to Fisher’s z values. At the group level, individual connectivity maps were entered into a one-sample t-test to identify brain regions showing reliable positive connectivity with the seed. Only positive functional connectivity was considered, given the ongoing debate regarding the biological interpretation of negative connectivity (46, 47). The resulting group-level t maps were thresholded using voxel-wise false discovery rate correction at *p* < 0.05 and then binarized. Binarized maps from all contrasts were overlaid to generate a network probability map. This map was further thresholded at 60% spatial overlap, a criterion previously validated in FCNM studies (48, 49), to define the final IGE-related normative connectivity network. As the primary objective was to identify common networks associated with the reported abnormal regions, coordinates of both increased and decreased GM alterations were pooled in the main analysis. The 60% threshold was used as the primary descriptive overlap criterion rather than as an inferential statistical threshold. Because a coordinate-based permutation or spatially constrained null-model analysis was not performed, the FCNM procedure could not determine whether the resulting overlap exceeded chance expectation.

### Association with canonical brain networks

To facilitate biological interpretation, we evaluated the spatial relationship between the identified IGE-related network and eight canonical brain networks. The seven cortical networks—comprising the visual network, somatomotor network (SMN), dorsal attention network (DAN), ventral attention network (VAN), limbic network (LN), frontoparietal network (FPN), and default mode network (DMN)—were defined based on the Yeo et al. parcellation (50). The subcortical network was delineated using the Human Brainnetome Atlas (51) and encompassed the amygdala, hippocampus, thalamus, and basal ganglia (including the caudate, putamen, globus pallidus, and nucleus accumbens). We quantified observed spatial overlap by calculating the percentage of voxels within each canonical network that intersected with the primary descriptive FCNM map.

### Descriptive reproducibility and sensitivity analyses

To assess descriptive reproducibility and sensitivity to analytic choices, we replicated the FCNM analyses using the independent validation dataset (the cross-scanner SALD). We then conducted the FCNM analysis again using spheres with radii of 1-mm and 7-mm to assess the impact of seed size. Furthermore, based on the HCP database, we analyzed brain regions showing increases and decreases in GM using the FCNM method, with a fixed sphere radius of 4 mm. Due to limited prior research, subgroup validation analysis was performed only in JME patients. To evaluate the sensitivity of the observed spatial pattern to the overlap criterion, we repeated the FCNM procedure using 50 and 70% thresholds in addition to the primary 60% threshold. Finally, we performed a leave-one-contrast-out sensitivity analysis. One contrast was excluded at a time, and the FCNM analysis was repeated using the remaining 17 contrasts to generate a new FCNM map. This procedure was repeated 18 times, with each iteration corresponding to the exclusion of one contrast. These analyses evaluated descriptive reproducibility and sensitivity to analytic choices. They did not provide a formal test of whether the observed spatial overlap exceeded chance expectation or whether the identified pattern was disease-specific.

## Results

### Included studies and sample characteristics

After a systematic literature search and screening (Supplementary Figure S1), the 18 included studies yielded 18 independent contrasts and 111coordinates, including coordinates reflecting both decreased and increased GM. These studies comprised 1,492 participants, including 694 patients with IGE syndromes and 798 HCs (Table 1). The epilepsy subtypes investigated included JME, IGE, absence epilepsy, CAE, juvenile absence epilepsy, and GTCA, JME was the most frequently studied subtype. The mean age of patients ranged from 4.5 to 44.2 years, while that of HCs ranged from 5.1 to 43.5 years. Disease duration varied across studies, ranging from 0.3 to 20.2 years where reported.

**Table 1:** Sample and imaging characteristics of the studies included in the study.

Study	Type	No. of subjects (F)	Mean age (year)	Disease duration (year)	medication status	Scanner field strength (T)	Threshold	Coordinate space	Foci
Woermann et al. (17)	JME HC	20 (12) 30 (16)	44.2 ± 11.0 43.5 ± 5.4	NA	12 patients used antiepileptic drugs	1.5	*p* < 0.05, corrected	Talairach	1
Betting et al. (18)	JME AE GTCA HC	44 (30) 24 (14) 15 (8) 47 (23)	32.0 ± 9.0 27.0 ± 12.0 29.0 ± 10.0 32.0 ± 14.0	12.0 ± 4.0 8.0 ± 4.0 12.0 ± 11.0	33 JME patients, 20 AE patients, and 4 GTCS patients used valproate; 7 GTCS patients used carbamazepine	2.0	*p* < 0.05, corrected	MNI	6
Chan et al. (19)	CAE HC	13 (12) 109 (57)	18.0 ± 9.0 29.0 ± 9.0	11.0 ± 11.0	12 CAE patients used antiepileptic drugs	3.0	*p* < 0.05, corrected	MNI	5
Kim et al. (20)	JME HC	25 (15) 44 (27)	22.7 ± 5.1 23.1 ± 4.3	6.1 ± 3.8	17 patients used valproate, 6 used lamotrigine, and 2 used both	1.5	*p* < 0.05, corrected	MNI	**7**
Filho et al. (21)	JME HC	54 (27) 30 (14)	27.7 ± 8.9 30.1 ± 9.0	14.4 ± 8.9	47 patients used antiepileptic drugs	1.5	*p* < 0.05, corrected	MNI	8
Lin et al. (23)	JME HC	60 (31) 30 (16)	26.6 ± 8.8 30.1 ± 9.0	13.6 ± 9.1	60 patients used antiepileptic drugs	1.5	*p* < 0.05, corrected	MNI	9
Huang et al. (8)	GTCS HC	31 (15) 37 (17)	25.9 ± 6.9 25.5 ± 3.9	8.4 ± 7.0	NA	1.5	*p* < 0.05, corrected	MNI	12
Liu et al. (7)	JME HC1 GTCA HC2	15 (12) 15 (12) 10 (3) 10 (3)	21.0 ± 4.0 21.0 ± 4.0 21.0 ± 4.0 21.0 ± 4.0	6.0 ± 4.0 6.0 ± 4.0	25 patients used antiepileptic drugs	1.5	*p* < 0.05, corrected	MNI	11
O’Muircheartaigh et al. (83)	JME HC	28 (16) 55 (27)	33.6 ± 10.1 30.1 ± 7.7	20.2 ± 10.3	27 patients used antiepileptic drugs	3.0	*p* < 0.05, corrected	MNI	2
Kim et al. (13)	IGE HC	50 (28) 50 (26)	26.0 ± 7.2 26.8 ± 5.6	10.2 ± 4.7	48 patients used antiepileptic drugs	3.0	*p* < 0.05, corrected	MNI	1
Kim et al. (12)	IGE HC	49 (23) 42 (20)	25.9 ± 7.2 27.0 ± 5.9	10.4 ± 6.1	30 valproate, 6 lamotrigine, 6 levetiracetam, 2 topiramate monotherapy, 2 valproate/lamotrigine, and 3 valproate/levetiracetam.	3.0	*p* < 0.05, corrected	MNI	1
Tondelli et al. (9)	JAE HC	24 (19) 24 (14)	26.3 ± 11.9 30.6 ± 5.4	12.9 ± 11.7	13 patients used sodium valproat	3.0	*p* < 0.05, corrected	MNI	12
Wang et al. (14)	CAE HC	20 (12) 20 (12)	8.7 ± 1.0 8.8 ± 0.9	1.9 ± 0.8	20 patients were first-episode and drug-naïve	3.0	*p* < 0.05, corrected	MNI	2
Wang et al. (10)	GTCS HC	14 (4) 29 (17)	4.5 ± 3.2 5.1 ± 2.2	2.4 ± 1.9	14 patients used antiepileptic drugs	3.0	*p* < 0.05, corrected	MNI	11
Zhang et al. (22)	JME HC	67 (38) 56 (36)	23.1 ± 5.9 26.8 ± 4.7	8.7 ± 5.8	67 patients used antiepileptic drugs	3.0	*p* < 0.05, corrected	MNI	13
Shin et al. (11)	IGE VPA−HC	81 (54) 120 (53)	29.0 ± 8.8 29.3 ± 8.2	13.2 ± 8.6	112 patients used VPA	3.0	*p* < 0.05, corrected	MNI	2
Deng et al. (15)	IGE HC	31 (10) 31 (11)	10.7 ± 2.9 10.5 ± 3.1	0.3 ± 0.3	31 patients were first-episode and drug-naïve	3.0	*p* < 0.05, NA	MNI	2
Tae et al. (58)	JME HC	19 (13) 19 (13)	22.6 ± 5.23 23.0 ± 7.33	7.9 ± 4.7	19 patients used antiepileptic drugs	1.5	*p* < 0.05, corrected	MNI	6

JME, juvenile myoclonic epilepsy; IGE, idiopathic generalized epilepsy; AE, absence epilepsy; CAE, childhood absence epilepsy; JAE, juvenile absence epilepsy; GTCS, generalized tonic–clonic seizures alone; GTCA, generalized tonic–clonic seizures alone; VPA, valproic acid; VPA-, VPA-untreated, valproate; HC, healthy control; NA, not available; MNI, Montreal Nneurological Institute.

### Observed normative functional connectivity associated with reported GM alterations in IGE

Using the HCP normative connectome, FCNM revealed that anatomically heterogeneous GM alterations reported in IGE were associated with a common observed distributed normative connectivity pattern. The principal nodes of this network were located in the bilateral middle temporal gyri (MTG), bilateral thalami, left superior frontal gyrus (SFG), and right putamen (voxel-wise FDR-corrected *p* < 0.05; network probability threshold ≥ 60%) (Figure 1A).

**Figure 1:**
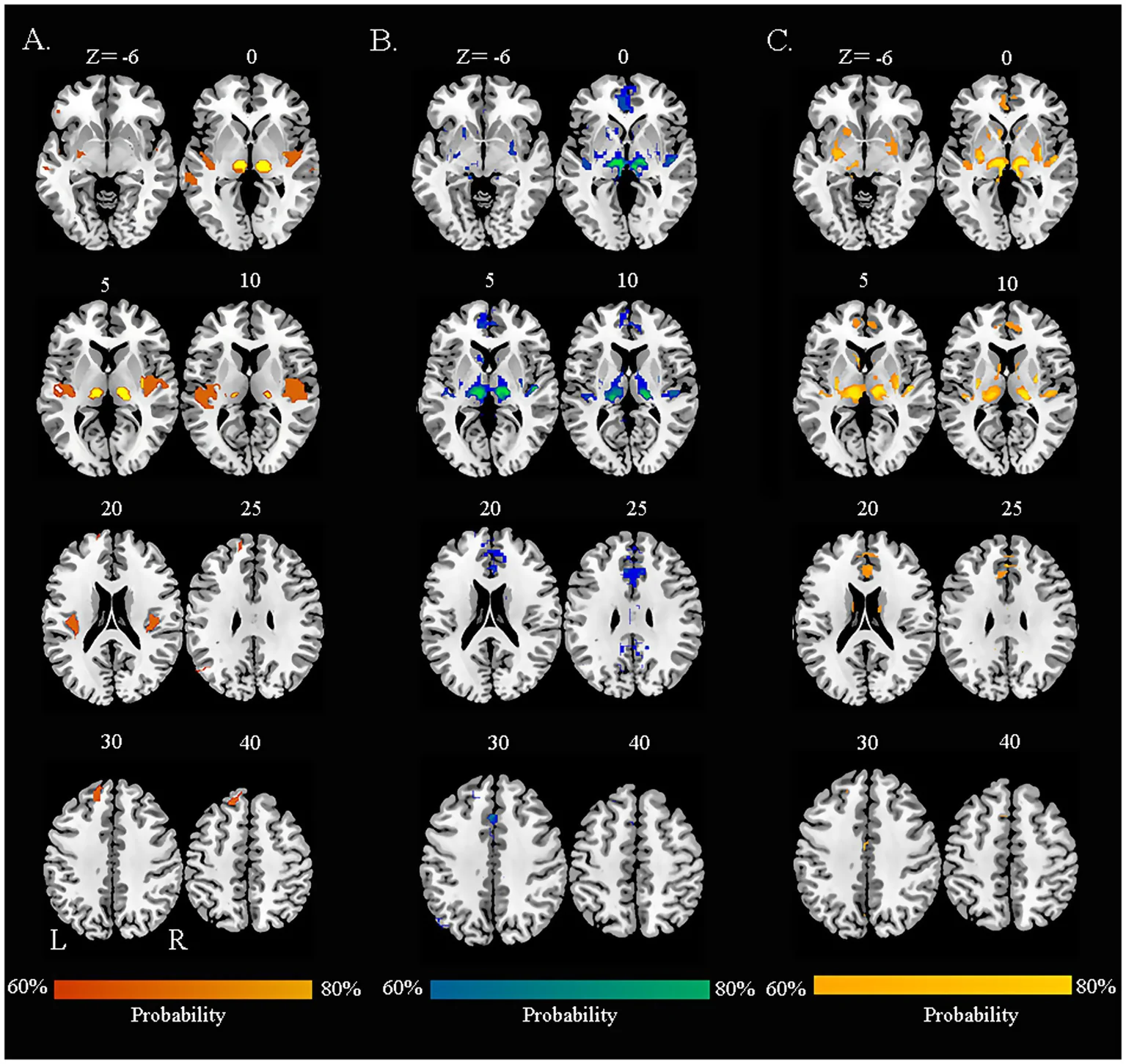
IGE-associated normative functional connectivity overlap maps generated using different seed radii. **(A)** 4-mm radius sphere; **(B)** 1-mm radius sphere; **(C)** 7-mm radius sphere. Observed IGE-associated normative connectivity patterns are shown as FC probability maps thresholded at 60%, representing brain regions functionally connected to at least 60% of the contrast seeds. IGE, idiopathic generalized epilepsy; FC, functional connectivity; L, left; R, right.

### Association with canonical brain networks

Spatial overlap analysis demonstrated that the IGE-related network was most strongly associated with the subcortical network, primarily involving the bilateral thalami and right putamen (overlap proportion = 42.77%). The second-largest observed overlap was observed with the DMN in the HCP discovery analysis; however, this association was modest and was less evident in the SALD validation analysis, mainly involving the bilateral MTG and left SFG (overlap proportion = 11.06%) (Figure 2A). Overlap with the visual, somatomotor, dorsal attention, ventral attention, limbic, and frontoparietal networks was below 10% for each network (Supplementary Table S3).

**Figure 2:**
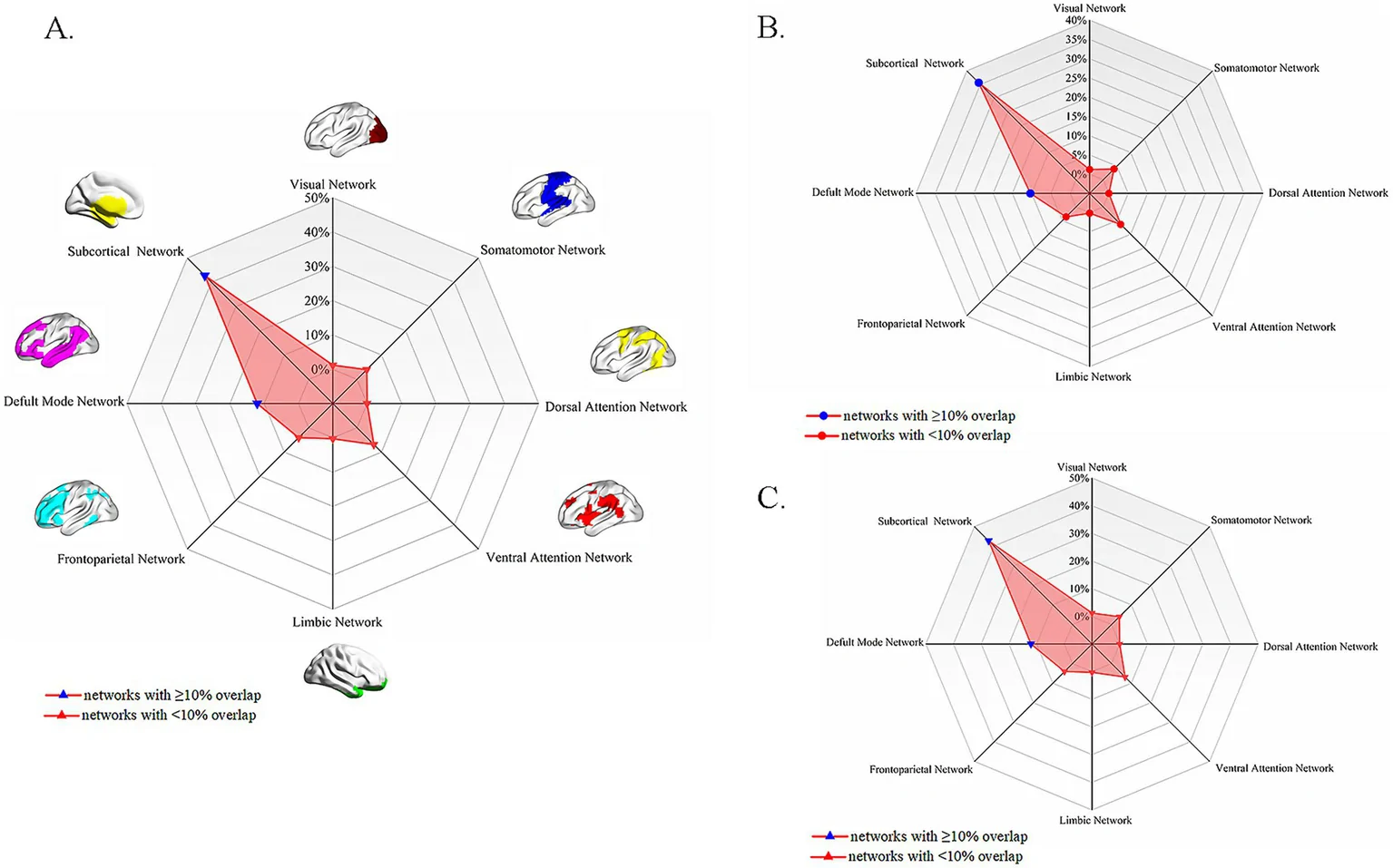
Observed spatial correspondence between the IGE-associated FCNM maps and canonical brain networks. **(A)** 4-mm radius sphere; **(B)** 1-mm radius sphere; **(C)** 7-mm radius sphere. Polar plots illustrate the proportion of overlapping voxels between each IGE map and a canonical network to all voxels within the corresponding canonical network. Note: blue dots indicate networks with an overlap proportion ≥10%, whereas red dots indicate networks with an overlap proportion <10%. IGE, idiopathic generalized epilepsy; FC, functional connectivity.

### Descriptive reproducibility and sensitivity analyses

Analysis using the independent SALD connectome yielded a broadly similar descriptive spatial pattern, with the most consistent convergence observed in subcortical regions (Supplementary Figure S2). Canonical network overlap analysis confirmed predominant correspondence with the subcortical network (overlap proportion = 43.98%), whereas DMN overlap was less pronounced than in the HCP discovery analysis (Supplementary Figure S3; Supplementary Table S3). Sensitivity analyses using 1-mm and 7-mm seed radii in the HCP dataset produced FCNM maps with broadly similar observed spatial distributions (Figures 1B,C) and preserved dominant overlap with the subcortical network and DMN (Figures 2B,C). We repeated the FCNM analyses separately for foci showing increased and decreased GM alterations to investigate potential direction-specific network patterns. The decreased-GM map showed the greatest observed spatial overlap with the subcortical network (overlap proportion = 65.84%), followed by the ventral attention network (16.33%). In contrast, the increased-GM map showed the greatest observed overlap with the DMN (37.23%) (Supplementary Table S4). Using the 50 and 70% overlap thresholds yielded FCNM maps with larger and smaller spatial extents, respectively, than the map based on the primary 60% threshold (Supplementary Figure S4). In the JME subgroup, the subcortical network showed an overlap proportion of 14.12% with the GM alteration map. Among the 18 leave-one-contrast-out analyses, 14maps (77.7%) reproduced the major network pattern identified in the primary analysis, characterized primarily by involvement of the subcortical network and the DMN (Supplementary Table S5).

## Discussion

In this study, we integrated coordinate-based VBM findings from the IGE literature with large-scale normative functional connectome data to characterize the normative connectivity patterns associated with heterogeneous GM alterations and to examine their observed spatial overlap with canonical brain networks. The principal descriptive finding is that reported GM alterations in IGE, although spatially dispersed at the anatomical level, were associated with a distributed normative connectivity pattern showing the greatest observed overlap with subcortical regions and additional DMN overlap in the HCP discovery analysis. Key nodes of this normative network included the bilateral thalami, right putamen, bilateral MTG, and left SFG. The subcortical component was replicated in an independent cross-scanner dataset and remained stable across alternative seed sizes, whereas DMN correspondence was less consistent across normative datasets. These results provide a network-level explanation for the inconsistent regional findings of prior VBM studies and support the view of IGE as a distributed cortico-subcortical network disorder. Importantly, because connectivity was derived from healthy participants, the identified network should be interpreted as the normative functional network connected to reported IGE-related GM alteration sites, rather than as direct evidence of abnormal functional connectivity in patients.

The DMN, especially the nodes of the MTG and SFG identified in our study, plays a crucial role in cognitive and emotional processing, including self-referential thinking, autobiographical memory, and emotional regulation (52, 53). Consistent with our localization, existing structural MRI and functional network studies have demonstrated that patients with JME may exhibit abnormalities in cortical morphology, GM structure, or functional connectivity in the MTG, suggesting that the MTG participates in disease-related alterations as an affected node within a distributed epileptic network (54–56). Abnormalities in GM concentration/volume in the SFG and adjacent prefrontal regions, impaired topological properties of structural covariance networks, and reduced hub status suggest that the SFG is an important component of epilepsy-related frontal network abnormalities (22, 57, 58). In the HCP discovery analysis, heterogeneous GM alterations in IGE showed secondary observed overlap with DMN regions, particularly the bilateral MTG and right SFG. However, this DMN correspondence was less evident in the SALD validation analysis and should therefore be interpreted as a less robust finding than the subcortical network localization. Previous studies have further demonstrated widespread and relatively consistent functional abnormalities within the DMN across multiple epilepsy syndromes, including JME, IGE, GTCA and absence epilepsy. The most consistently reported feature is altered functional connectivity in the posterior cingulate cortex/precuneus, a central hub of the DMN (59, 60). Beyond static connectivity alterations, recent studies have highlighted disruptions in the spatiotemporal dynamics of the DMN, including aberrant dynamic functional connectivity, altered temporal variability, and atypical state-transition frequencies and dwell time (61–63). Notably, dynamic analyses appear more sensitive than static approaches in capturing these subtle network anomalies. Studies using dynamic causal modeling and Granger causality analysis have additionally reported directional abnormalities in effective connectivity within the DMN (64, 65). Furthermore, additional studies have reported a dissociation between structure and function in the DMN of patients with GTCA, underscoring the heterogeneous effects of epilepsy on neural networks (66). Importantly, these intra-network alterations are tightly coupled with clinical phenotypes: longer disease duration and higher seizure frequency correlate with more pronounced DMN abnormalities (60, 63). Although the HCP discovery map showed 11.06% overlap with the DMN, this proportion was substantially lower than the 42.77% overlap observed for the subcortical network and was not comparably reproduced in the SALD dataset. We therefore interpret DMN involvement as a secondary and exploratory observation rather than a definitive network-level finding.

Beyond intra-network abnormalities, converging evidence indicates that epilepsy is also associated with disrupted interactions between the DMN and other large-scale brain networks. At the cortical level, patients with JME and other IGE syndromes exhibit attenuated anti-correlation between the DMN and the executive control network (ECN) (67). At the subcortical level, abnormal DMN–thalamus interactions may be among the most consistent findings. The thalamus is robustly engaged across the three principal seizure types associated with impaired consciousness—complex partial, generalized tonic–clonic, and absence seizures—and exhibits combined structural and functional connectivity abnormalities with the DMN (66, 68). Furthermore, the DMN displays anomalous interactions with the basal ganglia (linked to myoclonic motor manifestations), the SMN, the visual network (associated with photosensitivity), and the language network, reflecting the distributed nature of network-level disruption (64). Taken together, these findings are consistent with the conceptualization of epilepsy as a network-based disorder in which DMN dysfunction and aberrant inter-network interactions may jointly contribute to its pathophysiology. The evolution of analytic paradigms—from static to dynamic approaches, from single- to multi-network frameworks, and from functional connectivity to effective connectivity modeling—has deepened mechanistic understanding of epilepsy and may inform the future development of network-based biomarkers.

The subcortical network is broadly distributed and principally comprises the thalamus, caudate nucleus, and hippocampus (69, 70). It functions as a central integrative hub, primarily involved in motor control and coordination, integration and transmission of sensory information, and maintenance of arousal and consciousness (69, 71). Our finding that IGE-related GM alterations overlapped most strongly with this network is in line with the view that epilepsy—particularly secondarily generalized GTCA and JME—is more accurately characterized as a cortical–subcortical network disorder than as a condition driven solely by focal cortical abnormalities or diffuse whole-brain excitation (72, 73). Moreover, longitudinal neuroimaging studies have shown that progressive decoupling of cortical and subcortical networks, together with atypical subcortical hub reorganization, may underlie the synchronization of epileptic discharges and the emergence of cognitive and behavioral comorbidities (74, 75). Evidence from structural imaging, functional imaging, and neurophysiology provides convergent external support for a relationship between the thalamus and IGE. Structural MRI studies have shown that individuals with CAE, JME, and GTCA may exhibit abnormalities in total thalamic volume, regional thalamic morphology, or the volumes of specific thalamic nuclei (19, 76). In JME, structural abnormalities have been reported particularly in the anterior and inferior thalamic regions (77). In addition, patients with GTCA show reduced GM volume in the thalamus and frontal lobe (8). Functional imaging evidence further supports the active involvement of the thalamus in epileptic seizures, as thalamic activation has been observed during typical childhood absence seizures, coinciding with generalized spike-and-wave discharges (78). Neurochemical investigations have revealed changes in GABA levels in the frontal lobe and thalamus of individuals with JME (79). Beyond structural and neurochemical evidence, thalamocortical dynamics may link thalamic dysfunction to ictal impairment of consciousness across epilepsy syndromes. An intracranial EEG study in focal epilepsy showed that interictal thalamocortical network configuration recorded from the centromedian nucleus tracked susceptibility to consciousness-impairing seizures: patients without impaired consciousness showed relatively stronger thalamic synchronization with non-epileptogenic cortex, a differentiation lost in those with impaired consciousness, while secondary generalization was accompanied by reduced aperiodic activity in both the seizure-onset zone and the centromedian nucleus (80). Previous evidence further implicates the anterior and centromedian thalamic nuclei in epilepsy. Both exert broad control over cortical activity through diffuse reciprocal cortico-thalamo-cortical circuits, and their dysfunction contributes to seizure initiation and propagation in focal and generalized epilepsies. Accordingly, thalamic deep brain stimulation and vagus nerve stimulation likely act, at least in part, by restoring normative thalamocortical rhythms (81).

In the present study, subcortical correspondence was primarily driven by the bilateral thalami and right putamen. The putamen, a central nucleus of the basal ganglia, has been implicated as a subcortical hub in the pathogenesis of generalized epilepsies including JME, CAE, and GTCA (82–85). Multimodal neuroimaging studies have revealed microstructural integrity deficits in the putamen of individuals with epilepsy, as evidenced by elevated mean diffusivity and white matter tract injury (84–87). These structural alterations have been linked with abnormal dynamic functional connectivity within thalamo-cortico-striatal circuits (88). At the neurochemical and circuit-modulatory levels, diminished dopamine D2/D3 receptor binding in the putamen may reduce the inhibitory effect of the basal ganglia on pathological cortical discharges, potentially facilitating the propagation of generalized spike-and-wave discharges and the development of clinical seizures (89). Furthermore, progressive functional dissociation between the putamen and both prefrontal and sensorimotor cortical networks may relate not only to motor symptoms but also to higher-order cognitive impairments such as frontal executive dysfunction frequently observed in epilepsy (10, 75, 83, 87). The observed inclusion of the right putamen in the FCNM map is compatible with this striatal evidence. The direction-specific exploratory analyses suggested potentially different normative network patterns for increased and decreased GM alterations. Decreased-GM foci were predominantly associated with the subcortical network, whereas increased-GM foci showed greater correspondence with the default mode network. These findings may indicate that GM increases and decreases are not completely interchangeable and could reflect partly distinct neurobiological processes. However, the direction-specific analyses were based on fewer contrasts and should therefore be interpreted as hypothesis-generating rather than definitive.

Collectively, these findings offer a possible network-level framework for interpreting the spatial inconsistencies of prior VBM studies. The observed FCNM map showed greater overlap with the subcortical network and smaller, less consistently reproduced overlap with the DMN. However, the present analyses do not establish that these overlaps represent convergence beyond chance or two disease-specific systems. Although the present study does not directly identify treatment targets, the convergence of reported GM alterations onto thalamic and striatal networks may help generate hypotheses for future network-guided neuromodulation studies. Another major source of uncertainty is the developmental mismatch between the normative functional connectome and the included VBM samples. The HCP cohort comprised healthy adults aged 22–37 years, whereas the VBM studies included patients ranging from early childhood to middle adulthood. Although an age-stratified analysis was considered, only a small number of studies enrolled predominantly pediatric patients, yielding insufficient contrasts and coordinates for reliable FCNM analysis. Moreover, several studies reported age ranges spanning both pediatric and adult participants, meaning that classification based on study-level mean age could not accurately capture individual developmental stage. Accordingly, the present findings should be interpreted as FCNM patterns derived from an adult normative reference connectome, rather than direct evidence of age-invariant network topography across developmental stages. Replication with age-matched pediatric and adolescent normative connectomes is required.

## Limitations

When interpreting the findings of this study, several limitations should be considered. First, our analysis relied on coordinate-based data extracted from published studies. Peak coordinates serve as summary statistics and lack spatial detail compared with complete statistical maps or data from individual participants (30, 37). Second, we mapped networks connected to GM alterations in IGE using a normative connectome derived from healthy adults. Although this is standard practice in network mapping (34, 90) and prior evidence suggests that sample selection has minimal impact on network topography, employing a connectome from a sample more closely matched to IGE populations might yield slightly different results. In addition, many included IGE cohorts involved children or adolescents, whereas the normative connectomes used here were derived from adults. Developmental differences in functional network architecture may therefore influence network localization. Future studies using age-matched pediatric or adolescent connectomes are needed. Third, due to the limited number of available prior studies, we were unable to perform separate network localization for analyses of GM alterations across different epilepsy subtypes. Future studies with larger and more subtype-specific datasets are warranted to further investigate potential differences among distinct forms of epilepsy. Fourth, the input data were heterogeneous in terms of analytic methods and acquisition scanners. While FCNM aims to identify convergence, such heterogeneity may introduce noise and obscure subtle network effects. Fifth, methodological choices such as seed radius and the 60% overlap threshold may influence network definition. We did not construct a spatially constrained null distribution. Therefore, the present findings cannot determine whether the observed subcortical overlap exceeds chance expectation or whether it partly reflects generic properties of the normative connectome, particularly the broad connectivity of the thalamus and other highly integrative regions. Sixth, our analysis neither incorporated negative findings from the original studies nor weighted studies by sample size. Additionally, unaccounted-for confounders—including demographic variability, differences in scanning parameters, and variations in analytic procedures across studies—may have introduced residual effects that cannot be fully excluded. Continued efforts toward standardization, larger samples, and transparent reporting will be essential for progress in the field. Seventh, the broader age range in the SALD dataset could have influenced the variations observed between the HCP and SALD FCNM outcomes. Aging is linked to alterations in functional network structure, potentially elevating the diversity in brain network configuration among individuals. Therefore, the diminished reproducibility of cortical/DMN components in SALD may partially result from age-related variability in normative functional connectivity, as well as scanner and preprocessing variations. Future studies using age-matched normative connectomes or age-stratified analyses are warranted. Eighth, the HCP and SALD datasets had distinct initial preprocessing pipelines, with ICA-FIX denoising applied in HCP but not in SALD. ICA-FIX removes many structured noise components, including components associated with head motion, physiological noise, white matter, and cerebrospinal fluid signals (91, 92). Although additional nuisance regression of global, white matter, and cerebrospinal fluid signals was applied to the HCP data to reduce residual non-neuronal variance and to improve consistency with the SALD preprocessing strategy, these steps may partially overlap with variance components already attenuated by ICA-FIX. Therefore, we cannot fully exclude the possibility that differences in denoising strategy, including potential over-removal of shared variance, influenced resting-state functional connectivity estimates. This issue may partly explain why the subcortical component was reproducible whereas cortical/DMN overlap was less consistent across datasets. Given that nuisance regression and global signal regression can substantially affect functional connectivity estimates, and that their optimal use remains debated (46, 93), future studies using uniformly preprocessed connectomes and sensitivity analyses with and without additional nuisance regression are warranted. Finally, FCNM identifies normative networks connected to reported structural alteration sites and does not test whether these networks show abnormal functional connectivity in patients with IGE. Future studies combining patient-level structural MRI and rs-fMRI data are needed to validate the clinical relevance of the identified network.

## Conclusion

In summary, this study applied FCNM to large-scale normative connectome data to characterize the normative connectivity patterns associated with reported GM alterations in IGE. The reported VBM findings showed the greatest observed spatial overlap with a normative subcortical network involving the thalamus and putamen, whereas additional DMN overlap was observed primarily in the HCP discovery analysis. The subcortical component showed greater descriptive reproducibility across the HCP and SALD connectomes than the DMN component and was not entirely dependent on the selected seed size or any single contrast. However, because no coordinate-based null-model or permutation analysis was performed, these findings do not establish convergence beyond chance or disease-specific subcortical involvement. They should therefore be interpreted as descriptive and hypothesis-generating evidence that may inform future, formally validated studies of distributed cortical–subcortical network organization in IGE.

## Data Availability

The original contributions presented in the study are included in the article/Supplementary material, further inquiries can be directed to the corresponding authors.

## Footnotes

1
https://www.sdmproject.com/utilities/?show=Coordinates

2
https://www.fil.ion.ucl.ac.uk/spm/

3
http://rfmri.org/DPABI
