# Supplementary material for: Normative functional network localization of gray matter alterations in idiopathic generalized epilepsy
Source: Front Neurol. 2026 Oct 15;17:1957963. doi: 10.3389/fneur.2026.1957963 (PMC13716609; doi:10.3389/fneur.2026.1957963)
Supplement: Supplementary file 1 [file Supplementary_file_1.docx]

**Supplementary materials**

**Normative functional network localization of gray matter alterations in idiopathic generalized epilepsy**

**Figure S1**. **Flow chart showing the process of study identification and exclusion.**

IGE, idiopathic generalized epilepsy; ROI, regions of interest; GM, gray matter; VBM, voxel-based morphometry.

**Figure S2. Descriptive IGE-associated normative functional connectivity map derived from the SALD connectome using 4-mm radius seeds.**

IGE-related normative connectivity networks are shown as FC probability maps thresholded at 60%, showing brain regions functionally connected to more than 60% of the contrast seeds.

IGE, idiopathic generalized epilepsy; FC, functional connectivity; L, left; R, right.

**Figure S3. Observed spatial correspondence between the SALD-derived FCNM map and canonical brain networks**.

Polar plots illustrate the proportion of overlapping voxels between each IGE map and a canonical network to all voxels within the corresponding canonical network. Note: blue dots indicate networks with an overlap proportion ≥10%, whereas red dots indicate networks with an overlap proportion <10%.

IGE, idiopathic generalized epilepsy; FC, functional connectivity.

**Figure S4.** **Spatial correspondence between the IGE-associated connectivity maps and canonical brain networks. (A)** thresholded at 50%. **(B)** thresholded at 70%.

IGE, idiopathic generalized epilepsy.

**Table S1. Demographic information of the discovery and validation datasets.**

| **Dataset** | **Sample size** | **Age (years)** | **Gender (F/M)** |
| --- | --- | --- | --- |
| HCP | 1093 | 28.78 ± 3.69 | 594/499 |
| SALD | 329 | 37.81 ±13.79 | 207/122 |

Age is expressed as mean ± standard deviation (mean ± SD). Abbreviations: F, female; HCP, Human Connectome Project; M, male; SALD, Southwest University Adult Lifespan Dataset.

**Table S2. Resting-state fMRI parameters of discovery and validation datasets.**

| **Parameter** | **HCP** | **SALD** |
| --- | --- | --- |
| Scanner | 3.0T Siemens Trio | 3.0T Siemens Trio |
| Sequence | GRE-EPI | GRE-EPI |
| TR (ms) | 720 | 2000 |
| TE (ms) | 33.1 | 30 |
| FA (°) | 52 | 90 |
| FOV (mm²) | 208×180 | 220×220 |
| Matrix size | 104×90 | 64×64 |
| Slice thickness (mm) | 2 | 3 |
| Slice gap (mm) | 0 | 1 |
| Slices | 72 | 32 |
| Time points | 1210 | 242 |

FA, flip angle; fMRI, functional magnetic resonance imaging; FOV, field of view; GRE-EPI, gradient-recalled echo-Planar Imaging; HCP, Human Connectome Project; SALD, Southwest University Adult Lifespan Dataset; TE, echo time; TR, repetition time.

**Table S3. Observed spatial overlap proportions between IGE-associated FCNM maps and eight canonical brain networks in the HCP discovery and SALD validation datasets.**

| **Canonical network** | **HCP discovery,**  **4-mm seed (%)** | **SALD validation,**  **4-mm seed (%)** |
| --- | --- | --- |
| Visual network | 1.2% | 1.1% |
| Somatomotor network | 3.96% | 2.06% |
| Dorsal attention network | 0 | 0 |
| Ventral attention network | 6.77% | 7.61% |
| Limbic network | 0.2% | 0 |
| Frontoparietal network | 4.07% | 2.24% |
| Default mode network | 11.06% | 4.81% |
| Subcortical network | 42.77% | 43.98% |

IGE, idiopathic generalized epilepsy; HCP, Human Connectome Project; M, male; SALD, Southwest University Adult Lifespan Dataset.

**Table S4. Direction-specific FCNM findings for increased and decreased gray matter alterations.**

| **Analysis** | **Studies** | **Contrasts** | **Coordinates** | **Main network pattern** |
| --- | --- | --- | --- | --- |
| Decreased | 16 | 16 | 74 | Subcortical network  (65.84%)  Ventral attention network  (16.33%) |
| Increased | 8 | 8 | 27 | Default mode network  (37.23%) |

GM, gray matter; FCNM, functional connectivity network mapping.

**Table S5. Descriptive results of the leave-one-contrast-out sensitivity analysis of FCNM**.

| **Excluded contrast** | **Remaining contrasts** | **Main overlapping network(s)** | **Consistent with primary result** |
| --- | --- | --- | --- |
| 1 | 17 | subcortical network (41.23%)  default mode network (9.54%) | Yes |
| 2 | 17 | subcortical network (33.29%)  default mode network (6.24%) | Yes |
| 3 | 17 | subcortical network (39.79%)  default mode network (9.82%) | Yes |
| 4 | 17 | subcortical network (57.91%)  default mode network (9.53%) | Yes |
| 5 | 17 | subcortical network (39.03%)  default mode network (7.61%) | Yes |
| 6 | 17 | subcortical network (26.57%)  default mode network (6.58%) | Yes |
| 7 | 17 | subcortical network (40.52%)  default mode network (5.64%) | Yes |
| 8 | 17 | subcortical network (21.75%)  ventral attention network (8.56%) | No |
| 9 | 17 | subcortical network (43.52%)  default mode network (8.01%) | Yes |
| 10 | 17 | subcortical network (19.31%)  default mode network (6.32%) | Yes |
| 11 | 17 | subcortical network (23.56%)  somatomotor network (12.23%) | No |
| 12 | 17 | subcortical network (36.87%)  default mode network (7.21%) | Yes |
| 13 | 17 | subcortical network (21.36%)  default mode network (6.32%) | Yes |
| 14 | 17 | subcortical network (53.69%)  default mode network (9.98%) | Yes |
| 15 | 17 | subcortical network (15.63%)  ventral attention network (13.12%) | No |
| 16 | 17 | subcortical network (18.54%)  default mode network (5.89%) | Yes |
| 17 | 17 | subcortical network (39.23%)  default mode network (12.13%) | Yes |
| 18 | 17 | subcortical network (25.74%)  frontoparietal network (14.69%) | No |

FCNM, functional connectivity network mapping.
